# Study on the influence of school active break on children's physical activity level and classroom on-task concentration

**DOI:** 10.3389/fpubh.2026.1905069

**Published:** 2026-08-03

**Authors:** Guoliang Li, Wenbo Liu, Yiran Zhao

**Affiliations:** 1 School of Physical Education, Zaozhuang University, Zaozhuang, China; 2 Department of University Physical Education, Qingdao Huanghai University, Qingdao, China; 3 Department of Physical Education, Tianjin Guanyun Primary School, Tianjin, China

**Keywords:** active break, children, classroom on-task concentration, moderate-to-vigorous physical activity, physical activity, stratified randomized control

## Abstract

**Background:**

Children's sedentary, lack of activities and low attention in class are common problems in global public health and education. Long-term sedentary will reduce children's moderate-to-vigorous physical activity (MVPA), damage attention and executive function, and be detrimental to physical and mental health and academic performance. This paper explores the intervention function of structured active break.

**Methods:**

In this study, 1,248 students from grades 1–6 were enrolled in a stratified cluster randomized controlled trial, with individual classes defined as randomization and clustering correction units and grouped according to grade, gender and BMI. The intervention group had a 5-min structured in-class active break, while the control group maintained routine work and rest. The intervention period was 12 weeks. Activity data were collected by ActiGraph triaxial accelerometer, and concentration was evaluated by momentary time sampling and cancellation test. Generalized Estimating Equations (GEE) was used to correct the clustering effect, and 95%CI, Cohen's d and partial η^2^ were reported. The test level was α = 0.05, and Bonferroni multiple comparison correction was used for subgroup analysis.

**Results:**

After the intervention, the MVPA and light physical activity (LPA) in the intervention group increased by 18.6 min and 27.4 min, respectively, and the sedentary behavior duration decreased by 36.8 min, and the differences in various indexes and concentration related indexes were extremely significant compared with those in the control group (*P* < 0.001). Stratified analysis showed that children with lower grades, overweight and weak baseline concentration benefited more from intervention, and there was a significant three-way interaction effect (corrected *P* < 0.01), but there was no statistical difference between gender subgroups.

**Conclusion:**

Short-term structured active break in class can improve children's activity, reduce sedentary, and improve their concentration, which has a significant effect on junior, obese and attention-deficit students. This study is limited by a single-site sample, short-term follow-up, and a lack of physiological biomarker measurements, which restricts the generalizability of its conclusions. The related neural mechanism is still theoretical speculation and needs long-term multi-center research to prove it. This low-cost intervention can improve school-based health outcomes and classroom learning efficiency, and provide empirical evidence.

## 1 Introduction

Contemporary school-age children are facing profound health crises in their in-school living patterns. The normalization of sedentary behavior, lack of physical activity and declining classroom on-task concentration have formed dual global challenges in public health and education, which seriously threaten children's physical and mental health development and improvement of academic efficiency (1, 2). The World Health Organization (WHO) has clearly listed sedentary behavior as an “invisible killer” endangering the health of children and adolescents (3, 4). Statistics show that the average daily in-school sedentary behavior duration of most school-age children worldwide exceeds 8 h. Primary school students in China have an overall relatively high sedentary behavior duration, which is obviously beyond the academically recognized healthy and reasonable range (5, 6). Long-term sedentary behavior not only directly reduces participation opportunities for moderate-to-vigorous physical activity (MVPA), resulting in only a small number of primary school students meeting the recommended standard of 60 min of daily MVPA (7, 8), but also causes physical problems such as metabolic disorders, body fat accumulation and scoliosis. Studies have confirmed that children with daily sedentary behavior duration over 4.5 h face higher risks of scoliosis and increased obesity incidence, which become core inducements for high incidence of chronic diseases in childhood (9, 10).

From the perspective of cognitive development, the damage of sedentary behavior to children's brain function is more hidden and far-reaching. Neuroscience studies have shown that children's capacity for sustained on-task attention is limited to a window of 15–25 min. However, the existing classroom generally adopts 40–45 min continuous teaching mode with fixed static rest arrangement, which does not conform to the objective law of children's cognitive development (11). Children's sitting for a long time will slow down blood circulation in the brain, resulting in insufficient oxygen supply to the prefrontal cortex, and at the same time reduce the secretion level of brain-derived neurotrophic factor (BDNF), which ultimately impairs many core executive abilities such as concentration maintenance, behavioral inhibition, and flexible thinking (12). The existing basic research suggests that children with insufficient physical activity have shorter classroom on-task concentration time, higher attention error rate and lower task switching efficiency, creating a vicious cycle characterized by prolonged sedentary behavior duration, diminished classroom attention, and poor academic efficiency (13). Previous studies have suggested that children who are in lower grades, overweight and obese, and have low baseline concentration are more affected by sedentary, which aggravates the gap between education equity and health equity (14).

As a low-cost and easy-to-land campus intervention program, structured classroom active break has gradually attracted academic attention in recent years. Different from traditional static rest, structured classroom active break emphasizes embedding short-term and structured physical activities in classroom intervals, breaking the continuity of sedentary behavior through alternating movement and static state, and activating physiological and cognitive functions simultaneously (15). Research analyses indicate that three 5-min in-class active breaks per day can increase children's MVPA duration and improve classroom on-task concentration accuracy. Domestic pilot researches such as Happy 10 min and Sunny Break have also proved that break-time physical activities can effectively reduce sedentary exposure and improve learning efficiency (16). However, the existing research has some obvious limitations: First, most of the research samples are small, the intervention period is short, there is no high-level evidence of stratified cluster randomized control, and the clustering effect is not statistically corrected, which may lead to overestimation of the intervention effect. Second, most studies only focus on sports or attention indicators, but do not systematically analyze the synergistic changes between physical activity and classroom on-task concentration. Subgroup differences are mostly analyzed after the event, and there is a high risk of false positive without multiple comparison and correction. The methodological details of measurement tools such as accelerometer and classroom behavior observation are not disclosed enough, which leads to poor repeatability of the research; The mechanism is mostly directly related to neurobiological pathways such as BDNF and prefrontal activation. Due to the lack of support from biological indicators, this study has strong inferences.

Based on the teaching scene of primary schools in China, this study adopts a stratified cluster randomized controlled trial (the random unit is class cluster), corrects the class cluster correlation by GEE model, describes the whole measurement process of accelerometer and classroom behavior observation, and systematically analyzes the intervention effect of structured active break on primary school physical activity (MVPA, LPA, sedentary behavior duration) and classroom on-task concentration (concentration duration, accuracy rate, attention error rate and task compliance). Grade, BMI, baseline concentration and gender stratification analysis were set as exploratory analysis afterwards, and multiple comparisons were corrected by Bonferroni method. The research aims to fill the methodological and empirical gaps in the current field, objectively evaluate the health and educational benefits of this intervention model, provide scientific, accurate and scalable empirical evidence for school health promotion, classroom efficiency optimization and children's physical and mental health development, and help the implementation of the “Healthy China” strategy in campus scenes.

## 2 Data and methods

### 2.1 Study subjects and sampling design

Stratified cluster randomized controlled trial design was adopted, and classes were treated as cluster-level randomization units, targeting all in-school students from Grade 1 to Grade 6 in public full-time primary schools. Inclusion criteria: (1) aged 6 to 12 years old, in good physical health, free from severe cardiovascular and cerebrovascular diseases, motor system injuries as well as mental and neurological diseases, and capable of participating in routine physical activities normally; (2) fully attending scheduled courses at school without long-term absence or suspension from school; (3) guardians providing informed consent and signing written informed consent forms, and students voluntarily cooperating with the whole process of data collection. Exclusion criteria: (1) subjects with exercise contraindications or those explicitly advised by physicians to restrict physical activities; (2) subjects with missing data due to school transfer, long-term leave and other reasons during the intervention period; (3) subjects failing to meet the valid wearing duration standard of accelerometers or having obvious deviations in concentration evaluation data.

A total of 1,248 students from grade 1 to grade 6 were enrolled in this study. According to the classification of grade, gender and BMI, the subjects of each layer were divided into intervention group and control group by simple randomization method, including 621 in intervention group and 627 in control group. Calculate the intra-class correlation coefficient (ICC) = 0.12, and then use the generalized estimation equations (GEE) to set the exchangeable correlation matrix to correct the class clustering effect. There was no significant difference in baseline age, gender composition, BMI grade, baseline physical activity level and classroom on-task concentration between the two groups (*P* > 0.05), which was comparable. This research plan has been approved by Science and Technology Ethics Committee of Zaozhuang University, and the research is carried out in strict accordance with the ethical principles of Helsinki Declaration.

### 2.2 Intervention protocol and implementation procedure

The intervention lasted for 12 consecutive weeks. The intervention group and the control group maintained identical curriculum arrangement, teaching content, after-school physical activity schedule and campus daily schedule, with differences only in break modes.

Intervention group: Three sessions of 5-min structured in-class active breaks were conducted every day, fixed after the second morning class, the first afternoon class and the third afternoon class respectively, jointly organized and implemented by head teachers and physical education teachers. All active breaks adopted unified standardized contents, focusing on full-body, low-intensity and equipment-free dynamic activities, including four modules: Warm-up joint activities (2 min: circling and flexion-extension of neck, shoulders, wrists, knees and ankles); Simple dynamic stretching (1.5 min: upper limb stretching, lateral trunk stretching and calf stretching); Rhythmic stepping and jumping (1 min: in-place stepping and jumping jacks following fixed rhythm commands); Low-intensity group interactive games (0.5 min: high-five between pairs and simple physical interactions). The activity intensity was controlled at 3–6 METs. Teachers guided the whole process with uniformly recorded command audio to standardize movements, control exercise intensity and avoid intense confrontation and sports injury risks. Before intervention implementation, all teachers received two rounds of unified training on activity procedures, safety guidelines and standard commands, and only qualified teachers were allowed to participate in implementation. Standardized implementation records were filled in each time to record activity duration, participation rate and students' conditions.

Control group: Participants followed the original conventional school break mode. They were only allowed free activities, toilet use, drinking water and desk rest, without organized dynamic active breaks or additional structured physical activities. Students were free to take various leisure behaviors such as chatting, walking and simple games during breaks. All campus environments, teaching arrangements and daily schedules were consistent with those of the intervention group except structured active breaks, ensuring that the two groups differed only in intervention variables.

To guarantee intervention adherence, the research team carried out on-site supervision twice a week to record implementation frequency and duration. Timely reminders and supplementary training were provided for classes with adherence rate below 80%. In this study, intervention adherence was defined as the ratio of actual implemented activities to total planned activities at the class level. Classes with implementation rate no less than 80% were regarded as qualified in adherence, while those below the standard received focused supervision and retraining. The final overall intervention implementation rate reached no less than 92.3%.

### 2.3 Observation indicators and measurement tools

#### 2.3.1 Objective measurement of physical activity

ActiGraph wGT3X-BT triaxial accelerometer (ActiGraph Company, USA) was adopted, which was worn on the non-dominant hip. Sampling frequency is 100 Hz; The data processing epoch length is 15 s. The instrument has complete standard validity and test-retest reliability for children: many verification studies show that it is highly consistent with the double-standard water and gold standard in the calculation of energy consumption, steps and sedentary behavior duration of children aged 6–12, and the ICC can reach 0.86–0.99, which is widely used in the global large-scale cohort (National Health and Nutrition Examination Survey, NHANES) of children's physical activity epidemiology. The low-frequency extended filtering can accurately capture children's low-intensity fragmented activity signals, and the error is much lower than that of questionnaires and pedometry. Students wear it all the time during the waking period at school, and the effective wearing time per day is ≥10h, and they are continuously measured for 7 school working days; Criteria for judging non-wearing period: if the count value is 0 for 60 min, it is judged as non-wearing, and GGIR (Accelerometer Data Processing Package) software is used to automatically identify and eliminate non-wearing data. The intensity tangent point adopts Evenson MET tangent point for children (17).

Main measurement indicators are as follows:

- Moderate-to-vigorous physical activity (MVPA) duration:eshold at ≥3.0 metabolic equivalent of task (MET) to calculate daily cumulative in-school duration (min/d);
- Light physical activity (LPA) duration: adopt the range of 1.1–2.9 METs to calculate daily cumulative in-school duration (min/d);
- Sedentary behavior duration: defined as activities ≤ 1.0 METs with continuous sitting for no less than 1 min, and the daily cumulative in-school sedentary behavior duration (min/d) was calculated.

#### 2.3.2 Classroom on-task concentration assessment

The momentary time sampling method combined with cancellation test was adopted to evaluate classroom on-task concentration, and the evaluation was conducted once before the intervention and once at the end of the 12-week intervention respectively. All observers and classroom teachers were blinded to group allocation, and do not tell the class that it is intervention/control, so as to avoid subjective scoring bias.

- Momentary time sampling: Uniformly trained observers randomly selected three 10-min observation periods in core courses including Chinese and mathematics, and recorded students' concentration status every 15 s. The judgment criteria included looking at the blackboard or teachers, keeping pace with teaching rhythm, and having no irrelevant movements or conversations, so as to calculate the proportion of classroom on-task concentration duration.
- Cancellation test: The standard digital cancellation task was adopted with a time limit of 3 min to count concentration accuracy rate and attention error rate.
- Task compliance: Classroom task completion status was scored on a four-level scale by classroom teachers and quantified into task compliance scores.

### 2.4 Statistical analysis

All the data were analyzed by SPSS 26.0 statistical software, and the generalized estimating equations (GEE) (exchangeable correlation matrix) was used to correct the intra-group correlation and eliminate the clustering bias of students in the same class. The measurement data is expressed by the mean standard deviation, and the counting data is expressed by the number of examples (percentage). Paired *t* test was used for comparison before and after the intervention in the group, and GEE model was used for comparison between the groups to correct the baseline index and cluster effect. Repeated-measures ANOVA was applied to test time-by-group interaction effects to evaluate the interaction effect between time and grouping; Cohen's d was used in the unified report of effect size, and the repeated measurement model reported partial η^2^; Taking baseline age, gender, BMI and baseline indicators as covariables, the differences between the two groups were compared by analysis of covariance (ANCOVA). According to grade (junior/senior), BMI (normal/overweight and obesity), baseline concentration (high/low) and gender (male/female), stratified subgroup analysis was carried out. The multiple comparisons were corrected by Bonferroni method, and the test level after correction was α = 0.05/4 = 0.0125. Test the three-way interaction effect of interaction term time × grouping × subgroup; The main effect test level α = 0.05, *P* < 0.05 is statistically significant.

## 3 Results

### 3.1 Baseline characteristics and follow-up status of research subjects

A total of 1,248 primary school students from Grade 1 to Grade 6 were enrolled, including 621 in the intervention group and 627 in the control group. Finally, 1,194 valid participants completed the whole intervention and data collection, with an overall effective follow-up rate of 95.7%. As shown in Table 1, there were no statistically significant differences between the two groups in age, gender composition, grade distribution, BMI stratification, baseline physical activity and classroom on-task concentration indicators (all *P* > 0.05). The two groups had good baseline balance and were comparable. During the intervention, the overall implementation rate of structured active breaks in the intervention group was 92.3%, and no adverse events such as sports injuries and falls occurred throughout the research period.

**Table 1:** Comparison of baseline demographic characteristics and main indicators between two groups ($\bar{x}$ ± s).

Indicators	Intervention group (*n* = 621)	Control group (*n* = 627)	Mean difference (95% CI)	Statistical value	*P* value
Age (years)	8.57 ± 1.53	8.62 ± 1.50	−0.05 (-0.22, 0.12)	*t* = 0.592	0.554
Boys [*n* (%)]	328 (52.8)	335 (53.4)	—	*x*^2^ = 0.047	0.829
Lower grades (Grade 1–3) [*n* (%)]	311 (50.1)	314 (50.1)	—	*x*^2^ = 0.000	1
Overweight and obesity [*n* (%)]	127 (20.5)	131 (20.9)	—	*x*^2^ = 0.037	0.847
Baseline MVPA (min/d)	31.4 ± 4.6	31.1 ± 4.5	0.3 (-0.2, 0.8)	*t* = 1.135	0.257
Baseline LPA (min/d)	72.3 ± 8.5	71.9 ± 8.3	0.4 (-0.5, 1.3)	*t* = 0.901	0.368
Baseline sedentary behavior duration (min/d)	215.6 ± 18.2	214.9 ± 17.9	0.7 (-1.3, 2.7)	*t* = 0.694	0.488
Baseline proportion of concentration duration (%)	62.8 ± 7.1	63.1 ± 6.9	−0.3 (-1.1, 0.5)	*t* = 0.723	0.47
Baseline cancellation test accuracy rate (%)	78.5 ± 6.4	78.2 ± 6.6	0.3 (-0.5, 1.1)	*t* = 0.816	0.415
Baseline attention error rate (%)	12.6 ± 2.3	12.4 ± 2.4	0.2 (-0.1, 0.5)	*t* = 1.429	0.153
Baseline task compliance (score)	2.6 ± 0.5	2.5 ± 0.5	0.1 (-0.0, 0.2)	*t* = 0.984	0.325

### 3.2 Effects of active breaks on children's in-school physical activity levels

**Figure 1:**
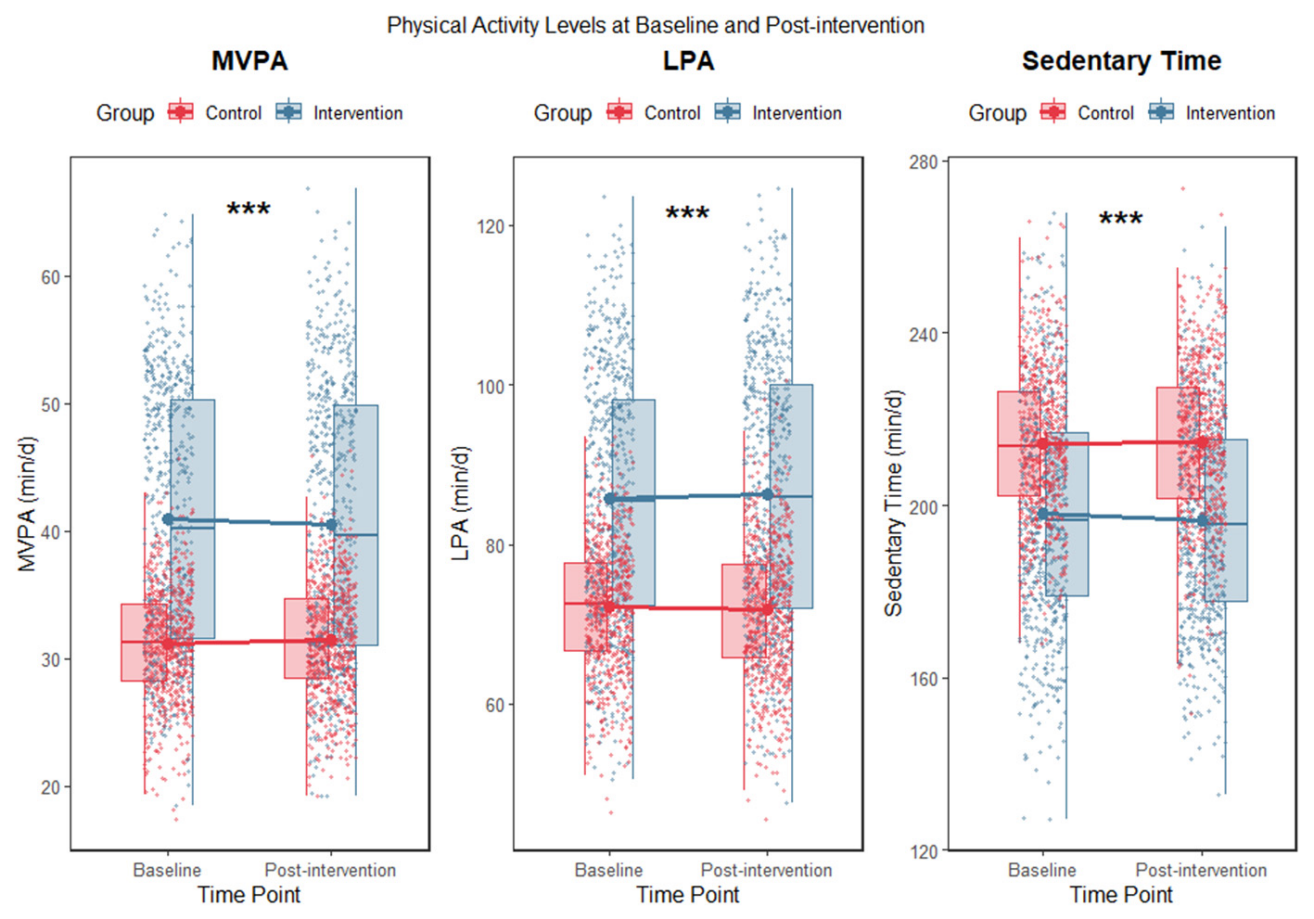
Changes in physical activity indicators in two groups of children before and after intervention. Note: Box plots combined with scatter plots show the distribution of daily moderate-to-vigorous physical activity (MVPA), light physical activity (LPA) and sedentary behavior duration of children in the control group (red) and intervention group (blue) before intervention and after 12-week structured active break intervention. The horizontal line inside the box represents the median, and the lines connecting box plots indicate the changing trend of mean values. ***P < 0.001, the interaction effect of time × grouping is significant, and the intervention after correcting class clustering can obviously improve the three physical activity indexes.

After 12 weeks of intervention, as shown in Figure 1, the levels of MVPA and LPA of children in the intervention group increased significantly, while sedentary behavior duration decreased markedly compared with baseline. No obvious changes were observed in all indicators of the control group before and after intervention. Repeated measures analysis of variance revealed significant time-by-group interaction effects (all *P* < 0.001). Analysis of covariance with baseline indicators as covariates indicated significant intergroup differences after intervention. The GEE model of physical activity indexes of children in the two groups before and after intervention corrected the class clustering and baseline variables, and the results of mean difference, 95% CI and effect size between the two groups are shown in Table 2.

**Table 2:** Changes in physical activity indicators in two groups of children before and after intervention ($\bar{x}$ ± s, min/d).

Index	Group	*n*	Pre–intervention	Post–intervention	Intra–group difference (95% CI)	Intra–group *t*–value	Intra–group Cohen's d	Adjusted between–group differences (95% CI)	Adjusted F value	partial η^2^	*P*–value
MVPA	Intervention group	621	31.4 ± 4.6	50.0 ± 5.3	18.6 (17.6, 19.6)	34.261	4.02	18.6 (17.2, 20.0)	186.43	0.131	< 0.001
	Control group	627	31.1 ± 4.5	31.5 ± 4.7	0.4 (-0.4, 1.2)	1.024	0.06	—	—	—	0.306
LPA	Intervention group	621	72.3 ± 8.5	99.7 ± 9.4	27.4 (25.6, 29.2)	29.105	3.08	27.4 (25.4, 29.4)	152.77	0.11	< 0.001
	Control group	627	71.9 ± 8.3	72.5 ± 8.6	0.6 (-1.0, 2.2)	0.715	0.07	—	—	—	0.475
Sedentary length	Intervention group	621	215.6 ± 18.2	178.8 ± 15.7	−36.8 (-38.9,−34.7)	−32.449	2.17	−36.8 (-39.5,−34.1)	171.39	0.123	< 0.001
	Control group	627	214.9 ± 17.9	213.5 ± 18.1	−1.4 (-3.7, 0.9)	−1.201	0.08	—	—	—	0.23

The exploratory results of stratified time period analysis show that the data of wearing in school for 10 h in this study can be seen after being split by course time period, and the increment of MVPA and LPA is highly concentrated in three standardized active break periods; There is no significant difference between the intervention group and the control group in the rest regular recess, class and physical education class time (*P* > 0.05), which proves that the improvement of physical activity comes directly from the compulsory organization activities of structured active break, rather than the uniform increase of students' independent activities throughout the day. As shown in Table 3, the intervention group achieved an average within-group daily MVPA increase of 18.54 min after the 12-week intervention. Subgrouped time-period analysis corrected for clustering effects further revealed that the between-group difference in MVPA solely generated from structured active breaks reached 11.92 min, which accounted for approximately 64.3% of the total within-group MVPA gain of the intervention group. The difference between the groups during this period was highly statistically significant (Wald χ^2^ = 3,842.71, *P* < 0.001), and the effect amount Cohen's d = 7.23, which was a great effect. However, in the other four non-intervention periods, such as regular recess, classroom teaching, physical education class and lunch break, there was no significant difference in the duration of MVPA between the intervention group and the control group, and the effect amount was in the small effect range (d = 0.07–0.22). The change of sedentary duration showed a highly consistent period specificity. The sedentary duration in the active break period intervention group decreased from 12.74 min in the control group to 0 min (the difference between groups was 12.74 min, *P* < 0.001), while the differences among the other four non-intervention periods were not statistically significant (*P* > 0.05). In terms of LPA index, there was no statistical difference in other periods except the active break period (0.82 min, *P* < 0.001).

**Table 3:** Comparison of physical activity indexes between groups at different periods after intervention.

Period classification	Physical activity indicators	Intervention group (*n* = 621)	Control group (*n* = 627)	GEE–corrected differences between groups	Wald χ ^2^	*P*–value	Cohen's d
		(min/d)	(min/d)	(95% CI) (min)			
Structured active break periods (3 times × 5 min, 15 min in total)	MVPA	12.84 ± 2.17	0.92 ± 0.68	11.92 (11.58, 12.26)	3,842.71	< 0.001	7.23
	LPA	2.16 ± 0.85	1.34 ± 0.72	0.82 (0.61, 1.03)	56.37	< 0.001	1.04
	Sedentary length	0.00 ± 0.00	12.74 ± 1.53	−12.74 (−12.93, −12.55)	15,876.29	< 0.001	−10.89
Regular recess period (Non–intervention class, about 35min)	MVPA	3.27 ± 1.04	3.05 ± 0.98	0.22 (−0.03, 0.47)	3.02	0.082	0.22
	LPA	14.83 ± 3.26	14.21 ± 3.15	0.62 (−0.11, 1.35)	2.89	0.089	0.19
	Sedentary length	16.90 ± 3.54	17.74 ± 3.62	−0.84 (−1.73, 0.05)	3.21	0.073	−0.23
Classroom teaching period (About 6 lesson hours × 40 min)	MVPA	1.53 ± 0.62	1.41 ± 0.59	0.12 (−0.02, 0.26)	2.68	0.102	0.2
	LPA	18.72 ± 4.15	18.05 ± 4.02	0.67 (−0.24, 1.58)	2.07	0.15	0.16
	Sedentary length	219.75 ± 16.83	220.54 ± 17.12	−0.79 (−2.87, 1.29)	0.57	0.45	−0.05
Physical education class period (3 class hours per week converted to daily average)	MVPA	8.56 ± 2.34	8.37 ± 2.28	0.19 (−0.42, 0.80)	0.54	0.462	0.08
	LPA	11.24 ± 2.87	11.08 ± 2.79	0.16 (−0.55, 0.87)	0.21	0.647	0.06
	Sedentary length	4.20 ± 1.35	4.55 ± 1.42	−0.35 (−0.76, 0.06)	2.91	0.088	−0.25
Lunch break and other school hours (about 90 min)	MVPA	3.80 ± 1.27	3.71 ± 1.21	0.09 (−0.25, 0.43)	0.42	0.517	0.07
	LPA	22.91 ± 5.03	22.52 ± 4.96	0.39 (−0.92, 1.70)	0.35	0.554	0.08
	Sedentary length	63.29 ± 7.64	63.77 ± 7.81	−0.48 (−1.88, 0.92)	0.46	0.498	−0.06
Full day in school Total (about 10 h)	MVPA	50.00 ± 5.30	31.46 ± 4.74	18.54 (17.14, 19.94)	687.42	< 0.001	3.74
	LPA	69.86 ± 9.42	67.20 ± 8.95	2.66 (1.12, 4.20)	11.63	< 0.001	0.29
	Sedentary length	304.14 ± 22.36	319.34 ± 23.51	−15.20 (−18.11, −12.29)	103.27	< 0.001	−0.66

### 3.3 Effects of active breaks on children's classroom on-task concentration related indicators

**Figure 2:**
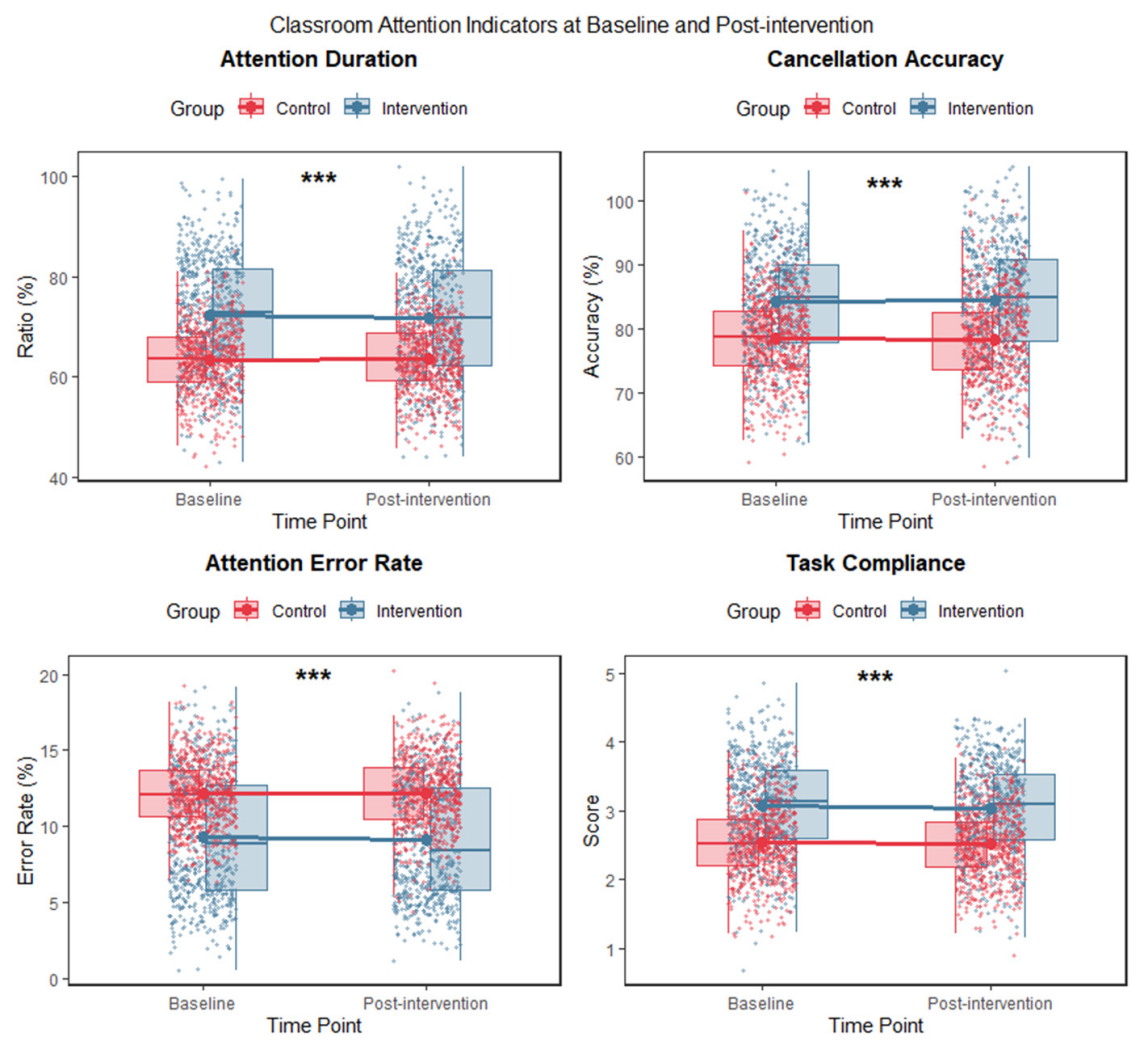
Changes of classroom on-task concentration indicators in two groups of children before and after intervention. Note: Box plots combined with scatter plots present the distribution of the proportion of classroom on-task concentration duration, cancellation test accuracy rate, attention error rate and task compliance of children in the control group (red) and intervention group (blue) before intervention and after 12-week structured active break intervention. The horizontal line inside the box stands for the median. *P < 0.001, time × group interaction effect is significant, suggesting that active break can improve classroom on-task concentration performance and reduce attention errors. ***Denotes a significant time-by-group interaction effect at *P* < 0.001.

After intervention, as shown in Figure 2, the proportion of classroom on-task concentration duration, cancellation test accuracy rate and task compliance scores in the intervention group were significantly increased, while the attention error rate decreased obviously. No remarkable changes were found in all concentration indicators of the control group. The intergroup differences were highly statistically significant (all *P* < 0.001). The specific changes of classroom on-task concentration indicators before and after intervention and intergroup comparison results of the two groups are shown in Table 4.

**Table 4:** Changes of classroom on–task concentration indicators in two groups of children before and after intervention ($\bar{x}$ ± s).

Index	Group	*n*	Pre–intervention	Post–intervention	Intra–group difference (95% CI)	*t*–value	Adjusted between–group differences (95% CI)	Adjusted F value
Percentage of Focus Time (%)	Intervention group	621	62.8 ± 7.1	81.3 ± 6.5	18.5 (17.2, 19.8)	27.642	17.6 (16.2, 19.0)	146.28
	Control group	627	63.1 ± 6.9	64.0 ± 7.0	0.9 (-0.3, 2.1)	0.917	—	—
Cancellation test accuracy (%)	Intervention group	621	78.5 ± 6.4	90.2 ± 5.7	11.7 (10.7, 12.7)	24.117	11.0 (9.8, 12.2)	119.54
	Control group	627	78.2 ± 6.6	78.9 ± 6.5	0.7 (-0.9, 2.3)	0.742	—	—
Attention error rate (%)	Intervention group	621	12.6 ± 2.3	5.8 ± 1.6	−6.8 (-7.4,−6.2)	−29.806	−6.3 (-6.9,−5.7)	165.72
	Control group	627	12.4 ± 2.4	11.9 ± 2.2	−0.5 (-1.1, 0.1)	−1.602	—	—
Task Compliance (Points)	Intervention group	621	2.6 ± 0.5	3.5 ± 0.4	0.9 (0.8, 1.0)	22.358	0.8 (0.7, 0.9)	105.17
	Control group	627	2.5 ± 0.5	2.6 ± 0.5	0.1 (-0.1, 0.3)	0.891	—	—

### 3.4 Results of stratified subgroup analysis

According to grade (junior/senior), gender (male/female), BMI (normal/overweight/obese) and baseline concentration (low/high), stratified subgroup analysis was carried out, and subgroup interaction analysis was carried out after the event. Bonferroni was used to correct multiple comparisons, and the corrected α = 0.0125. As shown in Table 5, children in lower grades, those with overweight and obesity, and children with low baseline concentration obtained more significant benefits in the improvement of physical activity and classroom on-task concentration, while the intervention effects showed no statistical differences between gender subgroups. Stratified subgroup analysis indicated that three-way interaction effect effects of time-group-grade, time-group-BMI and time-group-baseline concentration existed in MVPA, sedentary behavior duration, cancellation test accuracy rate and attention error rate (all *P* < 0.01). No statistically significant three-way interaction effects were found for all indicators in gender subgroups (all *P* > 0.05).

**Table 5:** Comparison of intervention effects of main outcome indicators among different subgroups.

Stratification factor	Subgroup	Indicators	F value	Corrected *p*–value	partial η^2^	Cohen's d
Grade	Lower grade	MVPA	98.74	< 0.001	0.142	1.12
			sedentary behavior duration	89.62	< 0.001	0.131	−1.05
			Cancellation accuracy rate	92.15	< 0.001	0.136	1.08
			Attention error rate	95.38	< 0.001	0.139	−1.1
	Higher grade	MVPA	27.41	< 0.01	0.042	0.48
			sedentary behavior duration	24.16	< 0.01	0.039	−0.45
			Cancellation accuracy rate	22.89	< 0.01	0.037	0.43
			Attention error rate	25.6	< 0.01	0.04	−0.46
Gender	Male	MVPA	1.32	0.251	0.002	0.53
			sedentary behavior duration	1.17	0.279	0.001	−0.5
			Cancellation accuracy rate	1.09	0.296	0.001	0.47
			Attention error rate	1.24	0.266	0.001	−0.51
	Female	MVPA	1.21	0.272	0.001	0.51
			sedentary behavior duration	1.08	0.299	0.001	−0.48
			Cancellation accuracy rate	1.14	0.286	0.001	0.49
			Attention error rate	1.19	0.276	0.001	−0.5
BMI	Overweight and obese	MVPA	87.33	< 0.001	0.128	1.04
			sedentary behavior duration	81.45	< 0.001	0.121	−0.98
			Cancellation accuracy rate	84.72	< 0.001	0.125	1.01
			Attention error rate	86.19	< 0.001	0.127	−1.03
	Normal weight	MVPA	29.06	< 0.01	0.045	0.51
			sedentary behavior duration	25.73	< 0.01	0.041	−0.47
			Cancellation accuracy rate	23.44	< 0.01	0.038	0.44
			Attention error rate	26.11	< 0.01	0.042	−0.48
Baseline concentration	Low level group	MVPA	92.6	< 0.001	0.135	1.09
			sedentary behavior duration	86.91	< 0.001	0.127	−1.02
			Cancellation accuracy rate	94.57	< 0.001	0.138	1.11
			Attention error rate	97.42	< 0.001	0.141	−1.13
	High level group	MVPA	8.37	< 0.05	0.015	0.27
			sedentary behavior duration	7.62	< 0.05	0.013	−0.25
			Cancellation accuracy rate	7.95	< 0.05	0.014	0.26
			Attention error rate	8.14	< 0.05	0.014	−0.26

## 4 Discussion

In this study, a stratified cluster randomized controlled trial with classes as random units was used, and the GEE model was used to correct the cluster correlation of classes. The intervention effect of structured active break on primary school students' physical activity level and classroom on-task concentration three times a day was systematically discussed. The results show that 12-week active break intervention can significantly increase children's high-intensity and light physical activity time in school, reduce sedentary exposure, and simultaneously improve classroom on-task concentration time, cognitive task accuracy and task compliance; After the exploratory stratified analysis, it is suggested that children with lower grades, overweight and obesity, and weak baseline attention have a higher benefit from intervention, and there is no significant difference in gender. This study improved the standardized methodological process of physical activity measurement and classroom behavior observation, reported the complete effect size and confidence interval, and provided methodological norms and empirical reference for the intervention of campus extracurricular activities; Due to the limitations of single-school samples, short-term follow-up and undetected biological markers, there is a boundary between causal inference and neural mechanism explanation in this study, and the relevant conclusions can only be cautiously extrapolated to similar primary school scenes.

### 4.1 Improving effect of structured active break on children's physical activity in school

This study confirms that short-term and high-frequency classroom active break can effectively improve children's physical activity patterns at school, which matches the conclusions of similar meta-analysis and randomized controlled trials in the world. At present, school-age children worldwide are generally faced with excessive sedentary behavior duration and substandard physical activity. A large proportion of children stay sedentary for more than 8 h per day, while less than 30% can reach the recommended standard of 60 min of daily moderate-to-vigorous physical activity (18). Long-term sedentary behavior directly leads to body fat accumulation, decreased cardiopulmonary function and abnormal spinal development, and continuously elevates the risk of metabolic syndrome, serving as a key behavioral inducement for the younger onset trend of chronic diseases in childhood (19–21). In this study, the average daily MVPA duration of children in the intervention group increased by 18.6 min/d, LPA duration rose by 27.4 min/d, and sedentary behavior duration decreased by 36.8 min/d, with highly significant intergroup differences. It indicates that even fragmented and low-intensity in-class activities can effectively interrupt continuous sedentary state and accumulate total physical activity volume. This result supports the sedentary interruption hypothesis proposed by modern exercise epidemiology, which holds that frequent breaks in sitting behavior bring greater health benefits than single long-duration exercise (22). It is found that inserting short-term activities can continuously reduce the cumulative injury caused by sedentary activities and increase the total amount of school activities throughout the day, which is the best intervention path for campus scenes (23). Compared with after-school physical activities and extended after-school services, in-class active breaks occupy no extra class hours, impose no additional site pressure and enjoy higher compliance. It is more suitable for large-scale promotion in primary schools with heavy academic pressure in China, and provides implementable behavioral intervention strategies to achieve the health promotion goals for children and adolescents under the Healthy China 2030 Initiative (24, 25). This study only collects physical activity data during school hours, and does not include family and off-campus activity indicators, which can not fully reflect the change of the total amount of activities throughout the day, and the conclusion has scene limitations.

In this study, the data of 10-h accelerometer in school were disassembled by time periods, and it was confirmed that all the increments of MVPA and LPA were concentrated in the 15-min organization period of standardized active break three times a day, and there was no statistical difference in the activity duration between the intervention group and the control group during the rest regular recess, classroom, physical education class and lunch break, which proved that the improvement of physical activity under short-term intervention first came from the increase of immediate activities brought by teachers' unified organization and instruction constraints, and was not the improvement of uniform spontaneous exercise throughout the day. Command-driven (short-term core action mechanism): During the entire experimental intervention cycle, teachers restrict students' independent and loose behavior through unified password guidance, standardized behavior flow control and on-site supervision throughout the process, put an end to negative states such as sitting in the classroom and emptying in a daze, and improve students' physical activity in a compulsory way. This is also the direct reason why the 12-week short-term intervention in this study can produce behavior increment. This behavior change pattern is highly consistent with the core characteristics of individual behavior change under external regulation in self-determination theory. Potential transfer effect of motivation and habit (long-term effect to be verified): After 12 weeks of short-term intervention, students' voluntary exercise behavior did not actively improve in off-campus and non-intervention scenarios, which indicates that short-term intervention has not helped students establish self-exercise habit, nor has it formed an intrinsic exercise pleasure drive. If the intervention period is extended to 6 to 12 months, so that students can continuously gain the experience of alternating static rest and dynamic exercise, it is expected that their exercise preferences will be gradually optimized, internal exercise motivation will be cultivated, and active exercise behaviors will be extended to daily scenes such as family and after-school. However, this inference of long-term behavioral transformation still needs to be further confirmed by longer-term follow-up studies. Mediating effect of physiological activation: Short-term phased exercise can activate students' cardiopulmonary function, relieve physical fatigue, effectively reduce the subjective adaptation threshold of students' classroom meditation, and reduce persistent sedentary behavior. On this basis, the total amount of light physical activity of students can be gradually accumulated, finally forming a benign behavior cycle of “breaking sedentary state, increasing physical activity and reducing sedentary time”. The improvement of physical activity in school brought by 12-week structured active break intervention is not the improvement of the uniformity of students' autonomous activity level throughout the day, but the 15-min active break period organized by standardized intervention; There is no significant change in the activity behavior pattern during the non-intervention period. The specific results of this period suggest that the increment of physical activity under short-term intervention is mainly contributed by immediate activities driven by teachers' unified organization and standardized process, and the behavior transfer effect to non-intervention periods such as regular recess and classroom interval has not been observed.

This study only collects the data of physical activity during the waking period at school, and does not include the acceleration monitoring at home, after school, after class and on weekends. There are two key limitations: First, it is impossible to completely evaluate the change of the total amount of 24-h activities and judge whether the intervention will have the activity compensation effect of “compensation for activities at school and increased sedentary outside school”, which has been observed in some campus intervention studies, weakening the overall health benefits throughout the day; Second, the lack of evidence of the mobility of extracurricular activities can not answer whether active break can cultivate autonomous exercise habits and extend to family scenes, and can only confirm the short-term immediate activity gain in campus scenes; Thirdly, it is impossible to control the interference of mixed variables such as extracurricular activity time, family exercise habits and parents' accompanying exercise on all-day activity indicators, which weakens the causal inference effectiveness of intervention to some extent. The existing research on active break in the same kind of campus generally has the limitation of only monitoring in school. In the future, it is necessary to adopt a 24-h wearable monitoring scheme to distinguish the increment of on-campus/off-campus activities and fully evaluate the all-day health effect of intervention.

### 4.2 Potential mechanisms of active break to improve concentration performance in the classroom

In terms of improvement of classroom on-task concentration, this study found that active break can significantly improve the proportion of children's concentration time, the correct rate of cancellation test, and reduce the rate of attention error. Its effect has clear cognitive function and physiological cycle explanation. From the perspective of physiological circulation, short-term moderate-intensity physical activity can rapidly increase the blood flow and oxygen supply of cerebral cortex, alleviate the insufficiency of blood supply to the brain caused by long-term meditation, and improve the basic activation level of cognitive control brain region (26, 27). As the core hub regulating sustained attention, inhibitory control and working memory, the prefrontal cortex presents significantly declined excitability under sedentary state, easily resulting in inattention, increased impulsivity and impaired executive function (28, 29). Short-term whole-body exercise can significantly improve children's prefrontal blood oxygen level, strengthen inhibition and control function, and reduce the rate of attention failure (30). The obvious drop of attention error rate among intervention group children directly reflects that active breaks improve the inhibitory control function of the prefrontal cortex. Explained by cognitive load theory, children have obvious age limitations in attention persistence, and the effective concentration window of children aged 6 to 12 is only 15–25 min. Traditional 40–45 min continuous classes tend to cause cognitive fatigue and attention exhaustion (31). Active break realizes the staged recovery of cognitive resources, reduces learning burnout and irrelevant behavior, and then improves classroom participation and task compliance through the rhythm reset of static and dynamic alternation, which is consistent with the educational neuroscience concept of “activities promote cognition” (32).

### 4.3 Practical implications of exploratory subgroup analysis

The stratified analysis of this study is an exploratory analysis after the event, and it is not written into the statistical analysis plan in advance. After Bonferroni's multiple comparison and correction, the intervention benefits of children with lower grades, overweight and obesity and low baseline concentration are more prominent, suggesting that active break has a precise protective effect on the health and cognitive disadvantaged subgroups. Previous studies have proven that overweight and obese children are generally characterized by low exercise willingness, strong sedentary tendency and poor cardiopulmonary endurance, accompanied by fluctuating attention and more classroom behavioral problems. Children in lower grades have immature nervous system and weak cognitive maintenance ability, making them more vulnerable to negative impacts of sedentary behavior. In this study, the above subgroups showed more prominent effects in increasing MVPA and reducing sedentary behavior duration, as well as greater improvements in classroom on-task concentration accuracy and error rate than other groups. These findings indicate that structured in-class active breaks may mitigate health and academic disparities driven by grade level, weight status, and baseline attention capacity to a certain extent, and play a positive role in promoting educational equity and health equity. This finding offers an important direction for targeted campus health intervention. When formulating break-time activity policies, priority can be given to children in lower grades, overweight and obese groups and those with poor attention to implement hierarchical guidance and targeted strategies, so as to maximize the benefits of public health investment.

### 4.4 Extension feasibility of intervention programs

The intervention model of this study is highly scalable. The daily three 5-min structured active breaks require no special equipment, do not adjust the original class schedule or increase extra workload for teachers. With mild activity intensity, it can effectively avoid sports injuries and classroom disorder, and is applicable to regular implementation in all kinds of primary schools in urban and rural areas. Compared with high-intensity physical activities, low-to-moderate-intensity full-body mild exercises are more acceptable for children and easier for head teachers to organize uniformly, thus ensuring long-term compliance. During the 12-week intervention period, the overall implementation rate reached 92.3% without any adverse events, further verifying that this scheme is safe, feasible and efficient. Against the background of the Double Reduction Policy, schools need to guarantee academic quality as well as fulfill health promotion requirements. The active break mode perfectly balances cognitive benefits and health benefits, realizing the dual goals of promoting learning and safeguarding health via physical activity.

### 4.5 Limitations

Several limitations still exist in this study. Only one public primary school in a single area is selected to carry out the investigation, and the sample representation is insufficient, so the extrapolation of conclusions is limited; The initial research design did not make it clear that the class was a random cluster and did not make cluster correction. Although GEE and ICC completed the post-event correction, the limited class sample size restricted the fitting of complex multi-layer model. There is still subjective bias in classroom behavior observation and teachers' evaluation. The accelerometer only collects data of 10 h' activities at school, and lacks 24-h activities at home, after class and on weekends, so it is impossible to evaluate whether there is compensation for off-campus activities in the intervention, and it is also impossible to verify the migration of sports habits to off-campus. No biological indexes of blood and brain function were detected; The intervention is only implemented for a short period of 12 weeks, and it is difficult to verify the persistence of the effect without long-term follow-up data; The stratified subgroup analysis is an exploratory analysis without pre-registration, and there is still the possibility of false positive after Bonferroni correction. At the same time, the mixed factors such as extracurricular sports and family work and rest are not controlled, which weakens the effectiveness of causal inference.

### 4.6 Future research outlook

Subsequent multi-center stratified cluster randomized controlled trials can be carried out to expand the samples of schools in urban and rural areas and different types of schools; Extend the follow-up period to 6~12 months, and follow up the long-term maintenance effect of intervention; The 24-h activity data of school and family were collected by wearing accelerometer synchronously, and the indicators of all-day activity in school intervention period, regular recess and off-campus were disassembled in different periods, and the total amount of 24-h activity behavior and compensation effect were evaluated completely. Biological indexes such as serum BDNF and near infrared brain imaging were included to directly verify the neuromolecular pathway of exercise improving attention; Pre-registration of hierarchical subgroup analysis scheme can reduce multiple comparative bias and provide a more stable and popularized scientific basis for campus inter-class intervention policy.

## 5 Conclusion

To sum up, structured in-class active breaks can significantly improve primary school students' physical activity level, reduce sedentary exposure, and simultaneously improve classroom on-task concentration and reduce attention error rate, especially for junior, overweight and obese children with low baseline concentration. This model is safe, simple and easy to popularize, which has both health benefits and educational value, and can be used as a conventional strategy for campus health promotion and classroom teaching optimization. Due to the limitations of single school sample, short-term follow-up, only collecting physical activity data in school, unable to evaluate 24-h activity compensation and habit transfer, unmeasured biological indicators, and after-the-fact subgroup analysis, the conclusions of this study can only be extrapolated cautiously, and the related neurobiological mechanism still needs to be further verified by multi-dimensional empirical research.

## Data Availability

The original contributions presented in the study are included in the article/supplementary material, further inquiries can be directed to the corresponding author.
